# Brain Inspired Sequences Production by Spiking Neural Networks With Reward-Modulated STDP

**DOI:** 10.3389/fncom.2021.612041

**Published:** 2021-02-16

**Authors:** Hongjian Fang, Yi Zeng, Feifei Zhao

**Affiliations:** ^1^Research Center for Brain-Inspired Intelligence, Institute of Automation, Chinese Academy of Sciences, Beijing, China; ^2^School of Future Technology, University of Chinese Academy of Sciences, Beijing, China; ^3^Center for Excellence in Brain Science and Intelligence Technology, Chinese Academy of Sciences, Shanghai, China; ^4^National Laboratory of Pattern Recognition, Institute of Automation, Chinese Academy of Sciences, Beijing, China

**Keywords:** brain-inspired intelligence, spiking neural network, reward-medulated STDP, population coding, reinforcement learning

## Abstract

Understanding and producing embedded sequences according to supra-regular grammars in language has always been considered a high-level cognitive function of human beings, named “syntax barrier” between humans and animals. However, some neurologists recently showed that macaques could be trained to produce embedded sequences involving supra-regular grammars through a well-designed experiment paradigm. Via comparing macaques and preschool children's experimental results, they claimed that human uniqueness might only lie in the speed and learning strategy resulting from the chunking mechanism. Inspired by their research, we proposed a Brain-inspired Sequence Production Spiking Neural Network (SP-SNN) to model the same production process, followed by memory and learning mechanisms of the multi-brain region cooperation. After experimental verification, we demonstrated that SP-SNN could also handle embedded sequence production tasks, striding over the “syntax barrier.” SP-SNN used Population-Coding and STDP mechanism to realize working memory, Reward-Modulated STDP mechanism for acquiring supra-regular grammars. Therefore, SP-SNN needs to simultaneously coordinate short-term plasticity (STP) and long-term plasticity (LTP) mechanisms. Besides, we found that the chunking mechanism indeed makes a difference in improving our model's robustness. As far as we know, our work is the first one toward the “syntax barrier” in the SNN field, providing the computational foundation for further study of related underlying animals' neural mechanisms in the future.

## 1. Introduction

The human capacity for language is unique on the earth: although most animals communicate, only humans show this unbounded expressive power (Fitch, 2018; Jiang et al., 2018). A significant topic for cognitive neuroscience is determining how human computational capacities differ from those of other animals (Deacon, 1998; Matsuzawa, 2013; Dehaene et al., 2015a; Yang, 2016; Jiang et al., 2018). Previously, the generative algorithms acquired by animals seem mainly restricted to the lowest level of the Chomsky hierarchy (Chomsky, 1957, 1965)—that is, regular languages (Fitch and Friederici, 2012; Fitch, 2014; Jiang et al., 2018). Thus, it has often been proposed that a pivotal gap lies between the levels of regular or “finite-state” grammars, which are accessible to nonhuman animals, and supra-regular grammars or “phrase-structure” grammars, which may only be available to humans (Hauser et al., 2002; Fitch, 2014; Jiang et al., 2018).

Some researchers attempt to teach animals understanding symbol sequences with nested or recursive structures, which are characteristic of human languages, have mostly been met with negative results (Miles, 1990; Pinker, 2003; Dehaene et al., 2015b).

So far, the generative algorithms acquired by animals seem mostly restricted to the lowest level of the Chomsky hierarchy (Chomsky, 1957, 1965)—that is, regular languages (Fitch, 2004; Fitch and Friederici, 2012). Thus, it has often been proposed that a “syntax barrier” lies between the levels of regular or “finite-state” grammars, which are accessible to nonhuman animals, and supra-regular grammars or “phrase-structure” grammars, which may only be available to humans (Hauser et al., 2002).

However, Jiang et al. (2018) designed the macaque monkeys supra-regular rule experimental paradigm, and they demonstrated that after extensive reinforcement training, macaque monkeys can master the supra-regular grammar, which breaks the barrier of syntax previously divided. Specifically, as Figure 1B in Fitch (2018) shown, Jiang and Wang designed a novel behavioral paradigm, delayed-sequence production task that required the animal to explicitly generate sequences according to the instructed grammars (Jiang et al., 2018). They compared two grammars:

(1) a “mirror” grammar of the form ABC|CBA, which in formal language theory involves recursive center embedding.

(2) a “repeat” grammar of the form ABC|ABC, i,e, repetition in serial order, as shown in Figure 1A in Jiang et al. (2018).

Like the grammars of all human languages, mirror grammars require a learner to possess supra-regular computational abilities, which requires specific computational machinery not needed at the lower sub-regular level Figure 1A in Fitch (2018). Besides, monkeys spontaneously generalized the learned grammar to novel sequences, including longer ones, and could generate hierarchical sequences formed by an embedding of two levels of abstract rules. Compared to monkeys, however, preschool children learned the grammars much faster using a chunking strategy (Jiang et al., 2018).

In fact, it is quite common for animals to complete the sequence by the “repeat” rule. The best example is that birds can imitate their parents' singing (Mooney, 2009). However, for the “mirror” sequence production, negative results are often obtained (Dehaene et al., 2015b). The essential reason is that most of the synapses (except electrical synapses) are unidirectional, and the reverse order production requires the agent to have the ordinal knowledge (Dehaene et al., 2015b). Even though when people are faced with the challenge of recalling a sequence of a phone number in reverse order, they often need to repeat the number sequence repeatedly to determine the position of a specific number in the sequence to complete the task. Therefore, the “mirror” sequence production task is a complex cognitive task that requires more advanced cognitive brain regions to participate in Fitch (2018). It is of great significance to reveal the cognitive process of reconstructing symbol sequence for understanding human language ability (Dehaene et al., 2015b; Jiang et al., 2018).

Their work inspired us to explore whether SNN can also break the “syntax barrier.”

After experimental verification, we demonstrated SNN could indeed handle the same sequence production task. The innovative aspects of this work are as follows:

- As far as we know, we are the first one to demonstrated that SNN can break the “syntax barrier” with Population-Coding and Reward-Modulated STDP mechanism, coordinating STP and LTP mechanisms simultaneously.
- We demonstrated that the chunking mechanism, helping to improve the robustness and learning efficiency of the network.
- Our work provides the computational foundation for further study of underlying animal neural mechanisms in the future.

## 2. Model and Methods

### 2.1. Neuron Model and Synapse Learning Rule

There are various neuron models such as the famous H- H model (Hodgkin and Huxley, 1952), Leaky Integrate-and-Fire neuron (*LIF*) model (Miller, 2018), Izhikevich neuron model (Izhikevich, 2003), and so on.

In order to simplify the computational complexity of the model, we choose the Leaky Integrate-and-Fire neuron model as the building block of the Spiking Neural Network to complete the whole experiment. Standard LIF models are shown in Equations (1), (2), and (3).

(1) $$\begin{array}{l} C_{m}\frac{dV}{dt}=-g\left(V-V_{s}\right)+I \end{array}$$

(2) $$\begin{array}{l} \tau_{m}\frac{dV}{dt}=-\left(V-V_{s}\right)+\frac{I}{g} \end{array}$$

(3) $$\begin{array}{l} V\to V_{reset}\;if\left(V\geq V_{threshold}\right). \end{array}$$

*C*_*m*_ is the membrane capacitance of the neuron, *V* is the membrane potential of the neuron, *g* is the conductance of the membrane, *V*_*s*_ is the steady-state leaky potential, here we let *V*_*s*_ = *V*_*reset*_ to simplify the model. *I* is the input current of the neuron. $\tau_{m}=\frac{C_{m}}{g}$ represents the voltage delay time, and different types of neurons have different values of τ_*m*_.

(4) $$\begin{array}{l} I=\underset{j}{\sum }w_{j,i}\sigma_{j}\left(t-1\right)+I_{s} \end{array}$$

(5) $$\begin{array}{l} \sigma_{i}\left(t\right)=\{\begin{array}{cc} 0 & V<V_{threshold}\\ 1 & V\geq V_{threshold} \end{array} \end{array}$$

Equation (4) shows that the current of neurons consists of two parts: the current from other neurons and the external stimulating current *I*_*s*_. *W*_*j,i*_ is the weight of i-th neuron to j-th neuron. σ_*i*_(*t*) is the indicator to judge if the i-th neuron firing at the time of t in Equation (5). And external stimuli mainly corresponding to the appearance of a specific symbol.

As for the synapse learning rule, Spike Timing Dependent Plasticity (STDP) (Bi and Poo, 1998; Dan and Poo, 2004) is one of the most important learning principles for the biological brain. STDP postulates that the strength of the synapse is dependent on the spike timing difference of the pre- and post-neuron (Dan and Poo, 2006).

Here we use STDP to update synaptic weights according to the relative time between spikes of presynaptic and postsynaptic neurons. The modulation principle is that if the postsynaptic neuron fires a few milliseconds after the presynaptic neuron, the connection between the neurons will be strengthened, otherwise, the connection will be weakened (Wittenberg and Wang, 2006). The update function is shown in Equation (6), where *A*_+_ and *A*_−_ are learning rates. τ_*s*_ and τ_*w*_ are STDP time constant, and Δ*t* is the delay time from the presynaptic spike to the post-synaptic spike.

(6) $$\begin{array}{l} \Delta w_{j,i}=\{\begin{array}{cc} A_{+}e^{\left(\Delta t/\tau_{+}\right)}\; & -\tau_{w}<\Delta t<0\\ -A_{-}e^{\left(-\Delta t/\tau_{-}\right)}\; & 0<\Delta t<\tau_{w} \end{array} \end{array}$$

All the parameters can be found in Table 1.

**Table 1:** Model parameters.

**Model/Rule**	**Parameter**	**Value**
LIF model	*C*_*m*_	30*nF*
	τ_*m*_	30 ms
	*V*_*reset*_	−65 mv
	*V*_*threshold*_	−35 mv
	τ_*ref*_	10 ms
STDP rule	τ_*s*_	15 ms
	τ_*w*_	10 ms
	*A*_+_	4
	*A*_−_	0.95

### 2.2. Working Memory Based on Population Coding

In the macaque monkeys' sequence producing experiment, researchers designed the paradigm where the macaque monkeys need to produce the sequence of the spatial symbols according to different rules, i.e., Repeat/Mirror.

Obviously, working memory is a necessary condition for sequence producing (Jiang et al., 2018). Just as the macaque monkeys must memorize the spatial symbols before producing process, our SNN should also include the corresponding circuit to accomplish working memory function. Therefore, we implemented the Working Memory Circuit (WMC) to realize related function, which will be covered in detail in this section.

Neurons can encode complicated temporal sequences such as the mating songs that songbirds learn, store, and replay (Quiroga, 2012; Yi et al., 2019). Inspired by the previous research work, an invariant, sparse, and explicit code, which might be important in the transformation of complex visual percepts into long-term and more abstract memories (Quiroga, 2012). It is reliable to assume when the macaques try to memorize the raw sequence, different populations of neurons are activated, i.e., they are bound to different light spots. Based on this assumption, we designed the Working Memory Circuit (WMC) to mimic the neuron activity of macaque monkeys.

**Figure 1:**
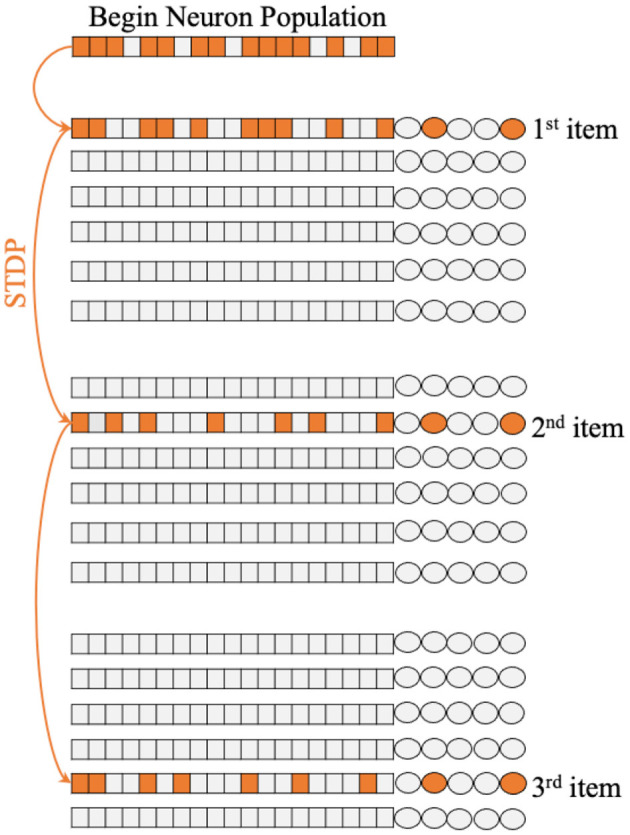
The architecture of Working Memory Circuit (WMC), each row of neuron populations corresponding to the six symbol on the screen shown to macaques in biology experiment. And the synapses between populations update with STDP learning rule.

Figure 1 shows the single unit of Working Memory Circuit, which includes six populations of neurons corresponding to six appear on the screen, the corresponding neuronal population will be stimulated in a short time window by an extra input current. Regarding the number of neurons of neuron population, we try different sizes in the experiment, which will be discussed in detail in the following chapters.

Inspired by biological discoveries we translate the appearance of single symbol in screen into the external input stimulation to corresponding spike neuron population. We choose Poisson Encoding as the method of input stimulation.

Due to the randomness of the Poisson Encoding, part of neurons in the population will fire at different times when the external stimulation window is given. The main function of inhibitory neurons is lateral inhibition. In order to make only one population of neurons fire among the six symbols, the inhibitory neurons in each population will inhibit remaining five populations of neurons.

Because symbols appear in sequential order, different populations of neurons will fire in turn. It is precisely because of different populations of neurons fire in a particular order, STDP rules can make a difference in the process of memory. Figure 1 shows how the STDP rules influence the memorizing process with different temporal activation of neuron populations.

The whole memorizing process starts with the cross in the center of the screen in Figure 1B in Fitch (2018) lit, which corresponds to the “begin” neuron population in WMC. This population obtains extra current and part of the memorize will fire. Then, according to the examples of Figure 1A in Jiang et al. (2018), symbols 1, 2, and 5 appear in turn, and the corresponding neuron population obtains the extra current in turn and fire in turn. Due to the mechanism of STDP, new synapses are formed between the corresponding populations of neurons of symbols 1, 2, and 5, as shown in Figure 1, then completing the memory process. It is worth mentioning that the “125” sequence is just an example for convenience of understanding, WMC can memorize the sequence composed of any three symbols in the set of position symbols.

### 2.3. Motor Circuit

In the macaques' sequence producing experimental paradigm, macaques need to press the light spots in the screen by correct sequence to get the reward (Jiang et al., 2018). Neuroscientists have found that in the biological brain, action instructions are encoded by specific motor neurons (Wichterle et al., 2002). Correspondingly, in our model, we must define the triggers of pressing action.

**Figure 2:**
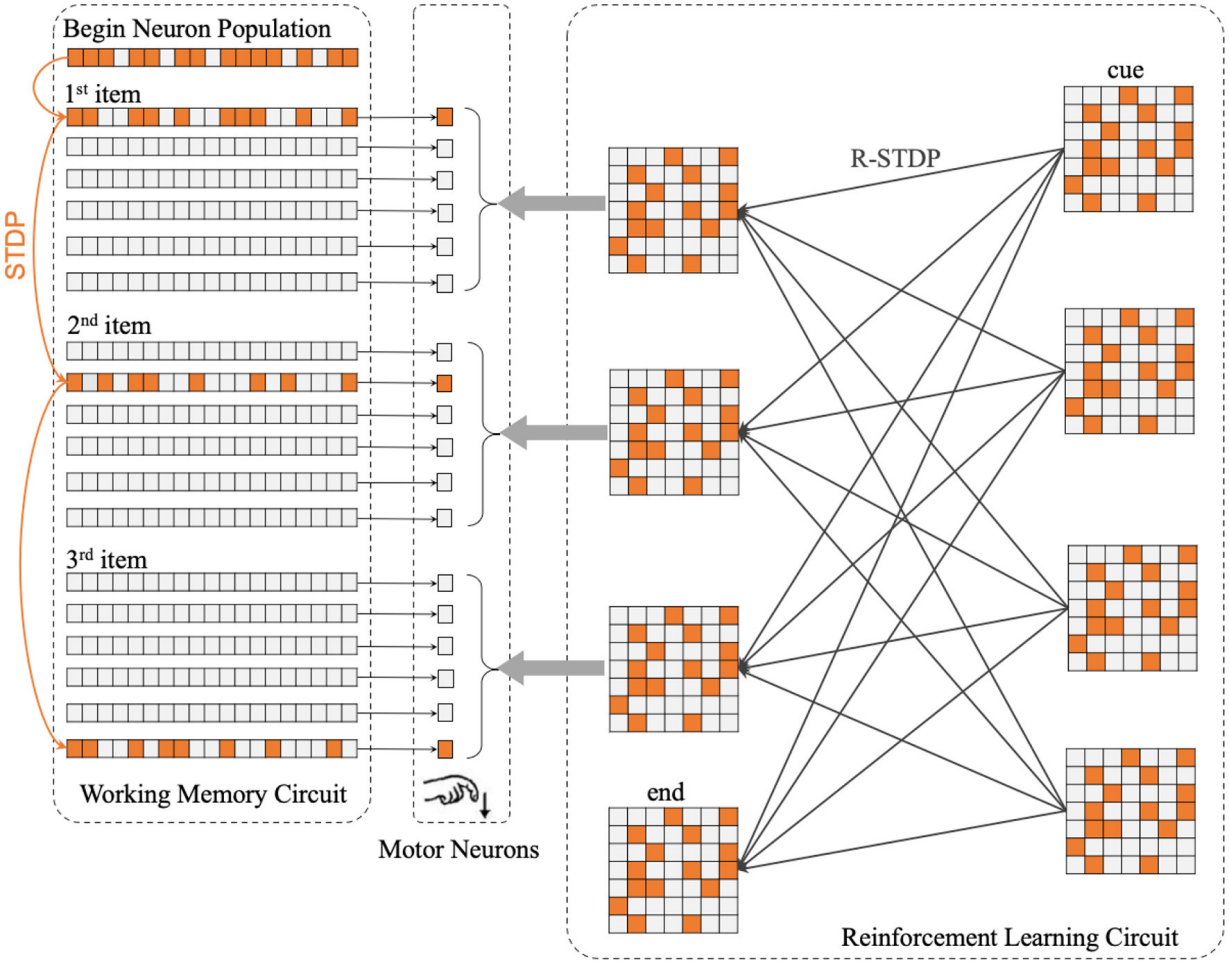
The whole architecture of SP-SNN is divided into three neuron circuits. The orange lines in Working Memory Circuit mean the synapses inner WMC that is formulated by STDP rule. The thin black lines between WMC and Motor Neurons represent every population neurons in WMC project to the same motor neuron, which fire to trigger the output action. The gray arrows between Reinforcement Learning Circuit and Motor Neurons display each population neurons in RLC project to the same motor neuron as well. Moreover, the thick black lines in RLC show the synapses inner RLC. With reinforcement learning, the network will gradually learn different sequence reconstruction rules, which will be reflected in the weight distribution of synaptic connections in RLC.

The center part of Figure 2 shows the concrete structure of the motor circuit. Motor circuit receives the projection from Working Memory Circuit and Reinforcement Learning Circuit.

In a nutshell the macaque monkeys perform a specific position keystroke operation once a corresponding motor neuron fire. In our SNN model, the network output a symbol once. (Six light spots, in this case, correspond to six motor neurons in each population).

### 2.4. Reinforcement Learning With Reward-Modulated STDP

Unlike the short-term plasticity (STP) (Markram and Tsodyks, 1996; Abbott et al., 1997; Zucker and Regehr, 2002) mechanism in the memory process, macaque monkeys use long-term plasticity (LTP) (Bi and Poo, 2001) mechanism as the mean of learning Repeat/Mirror rules (Jiang et al., 2018). That means macaque monkeys' memory about a particular sequence maintains short time, while the learning of producing rules are in the long term. According to the experimental paradigm in the references, macaque monkeys will be rewarded with food or water if they can complete the production of the sequence in the course of training, and punitive measures (i.e., blowing the monkey's eyes with air) will be launched if there are any symbolic errors in the production process (Jiang et al., 2018). Therefore, it is reasonable to assume that the learning of the Repeat/Mirror rules in macaque monkeys is based on reinforcement learning.

The Reinforcement Learning Circuit (RLC) on the right side of Figure 2 is the core function circuit that enables the network to master different rules. The RLC consists of presynaptic and postsynaptic parts, each of which contains several populations of neurons. In Figure 2, on the right side of RLC are the presynaptic neuron populations, which receive external stimulation and affect postsynaptic neuron populations in RLC; on the left side are the postsynaptic neuron populations, which receive the projection from the presynaptic neuron populations and transmit the signal to the motor neurons, guiding the motor neurons to fire in a specific order.

In addition to the presynaptic “cue” neuron population and postsynaptic “end” neuron population, the remaining six populations of neurons correspond to each other, divided into three population by row, corresponding to the first, second, and third positions in the sequence, respectively. Dehaene have proposed that a taxonomy of five distinct cerebral mechanisms for sequence coding: transitions and timing knowledge, chunking, ordinal knowledge, algebraic patterns, and nested tree structures (Dehaene et al., 2015b), which inspired us that the ordinal knowledge should be encoded by different populations of neurons.

Therefore, the core of the so-called different “rules” lies in the connection mode of the reinforcement learning circuit. Just as macaque monkeys acquire rules in experiments, the acquisition of rule learning of our networks is also a reinforcement learning process.

Before the experiment was completed in the macaque monkeys (Jiang et al., 2018), these two rules were considered supra-regular rules that only human beings could master. Figure 2 shows all the components of the network, including memory circuit, motor neurons population, and reinforcement learning circuit. In this case, for the convenience of introduction, we will describe the process to complete the sequences of length three production task.

How SNN can realize reinforcement learning is an open question hitherto, there is some leading research work in this field (Urbanczik and Senn, 2009; Frémaux and Gerstner, 2016; Wang et al., 2018). The main contradiction lies in the current learning rules of SNN synapses, such as STDP, Hebbian, etc., that the time of synaptic update was slightly later than the time of local neuron fire, however, in reinforcement learning, reward/punishment come after a trial. How to build a bridge between reward/punishment and synaptic learning rules such as STDP is where the crux lies (Izhikevich, 2007).

After full investigation, we adopt reward-modulated STDP (R-STDP) to implement the whole experiment due to the excellent biology plausibility (Frémaux and Gerstner, 2016).

The main idea of R-STDP is to modulate the outcome of “standard” STDP by a reward term (Friedrich et al., 2011).

**Figure 3:**
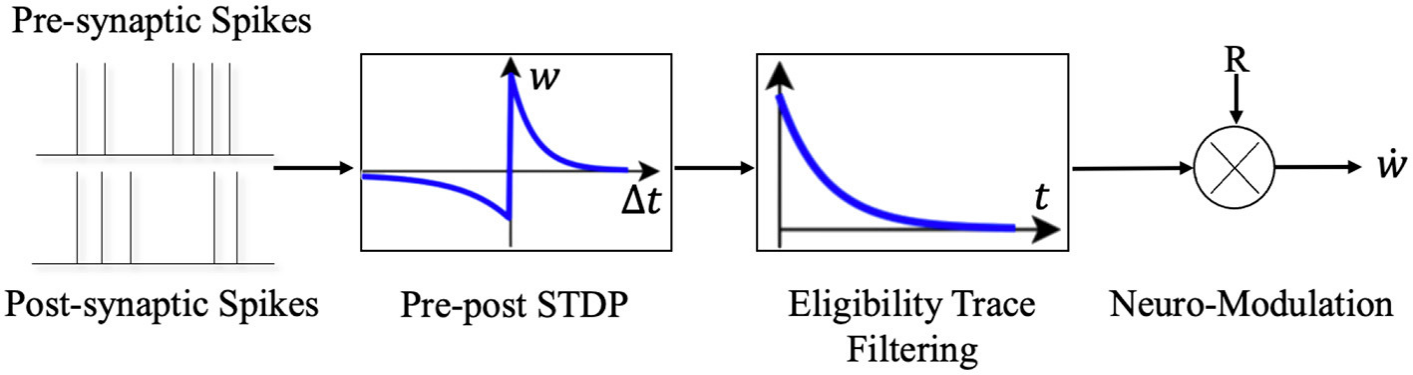
The schematic diagram of Reward-modulated STDP. Different from the general STDP rules, when neurons implements the R-STDP rules the synaptic weights will not be updated once the pre- and post-synaptic neurons generate spike pair, but temporarily stores the variations of weights in the eligibility trace. Only when the reward or punishment signal comes, the corresponding synaptic weights will be updated according to the current value of the eligibility trace and the reward/punishment signal.

Synaptic eligibility trace (right box in Figure 3) stores a temporary memory of the STDP outcome so that it is still available by the time a delayed reward signal is received (Frémaux and Gerstner, 2016). We regard the timing condition (or “learning window”) of traditional STDP as *STDP*(*n*_*i*_, *n*_*j*_), *n*_*i*_ and *n*_*j*_ denote the presynaptic and postsynaptic neuron in the network. The synaptic eligibility trace keeps a transient memory in the form of a running average of recent spike-timing coincidences. Synaptic eligibility traces arise from theoretical considerations and effectively bridge the temporal gap between the neural activity and the reward signal.

(7) $$\begin{array}{l} \Delta e_{j,i}=-\frac{e_{j,i}}{\tau_{e}}+STDP\left(n_{i},n_{j}\right) \end{array}$$

*e*_*j,i*_ is the eligibility traces between presynaptic neuron i and postsynaptic neuron j, τ_*e*_ is the time constant of the eligibility trace. The running average is equivalent to a low-pass filter. In R-STDP mechanism, the synaptic weight *W* changes when the neuromodulator *M* signals exist.

(8) $$\begin{array}{l} \Delta W=M*E \end{array}$$

Considering the complexity of the network, we simply choose R-max policy i.e., *M* = *R*. *R* is the reward or punish signal toward network which is given by the experiment environment. Actually, *R* is the function of time *t*, Equation (9) shows how *R* changes through time.

(9) $$\begin{array}{l} R\left(t\right)=\{\begin{array}{cc} C_{r}\; & t-t_{r}\leq T_{R}\\ C_{p}\; & t-t_{p}\leq T_{R}\\ 0\; & t-t_{r}>T_{R}\\ 0\; & t-t_{p}>T_{R} \end{array} \end{array}$$

*C*_*r*_ and *C*_*p*_ are the constants of reward and punish signal. *t*_*r*_ and *t*_*p*_ denote the latest time of reward and punish. And *T*_*R*_ is the size of time window of reward or punish signal. In the experiment, we set *C*_*r*_ = 10, *C*_*p*_ = −10, and *T*_*R*_ = 5.

Specifically, in the process of a sequence production, macaque monkeys need to memorize symbols sequence firstly, through STDP learning rules to complete the STP of WMC when the production process starts, under the guidance of the start signal, the neurons in WMC corresponding to the symbols in the original sequence fire in an ideal situation. Because there is a corresponding population to target connection between WMC and the motor neuron population, the membrane potential of motor neurons corresponding to the symbol increases. Although the membrane potential of these particular motor neurons has not reached the threshold of the action potential, it will be significantly increased compared with other neurons. Once the post-synapse neuron population in the RLC starts to fire frequently, the membrane potential of corresponding motor neurons population will rise quickly until fire. For a different rule, Repeat/Mirror, the network should produce the sequence by a different order, which means different firing order of post-synapse populations in RLC. It is where reinforcement learning makes a difference.

The more detailed learning process of SP-SNN is shown in Algorithm 1.

**Algorithm 1:** The learning process of SP-SNN

1. Initialize *N*_*population*_ = 50,*V*_*threshold*_ = −35*mv*, and other parameters of the network
2. Load Training Set(S)
3. Start training procedure
for every sequence in S do
Memory stage:
Increase *I*_*s*_ of corresponding populations
Update weights *W*_*WMC*_ with STDP rule by Equation (6) ⊳ STDP rule

Reinforcement learning stage:
Increase *I*_*s*_ of begin populations of RLC
if output correct sequence then
*R*(*t*) ← *C*_*r*_ ⊳ Give reward
else
*R*(*t*) ← *C*_*p*_ ⊳ Give punishment
end if
Update weights *W*_*RLC*_ by Equation (8) ⊳ R-STDP rule
end for
4. Start test procedure

### 2.5. The Chunking Mechanism of SP-SNN

Through the design and verification of the macaque monkeys supra-regular rule experimental paradigm, it is found that after intensive training, macaque monkeys can master the supra-regular grammar, breaking the barrier of syntax previously divided (Jiang et al., 2018). Jiang et al. (2018) point out that whether there is a clear boundary between human and animal language competence needs to be discussed in detail again (Fitch, 2018).

Neuroscientists and psychologists have been exploring the Chunking Mechanism for a long time (Ellis, 1996; Gobet et al., 2001; Fujii and Graybiel, 2003). It is generally believed that this mechanism plays an essential role in human short-term working memory (Burtis, 1982), knowledge acquisition (Laird et al., 1984; Gobet, 2005), and even skill learning (Rosenbloom et al., 1989; Pammi et al., 2004). Bibbig et al. (1995) showed that after learning the hippocampus neurons form chunks that are special representations for co-occurrence of neural events in several association areas via computer simulations of a spiking neural network.

Decomposing a long sequence into several shorter sequences to improve the efficiency and accuracy of memory is the core component of chunking mechanism (Ellis, 1996). For example, it is difficult for one person to remember a whole sequence of mobile phone numbers. Instead, the mobile phone number sequence is decomposed into several shorter sequences to realize the memory. Therefore, inspired by Chunking Mechanism in the cognitive process of biological brain, we try to explore the introduction of a biologically similar Chunking Mechanism into the network, and then observe the changes in network performance of sequence representation. Specifically, compared with the network without Chunking Mechanism, the main difference of the new network is the connection mode, i.e., after the introduction of Chunking Mechanism, the long sequence is segmented into several shorter chunks.

**Figure 4:**
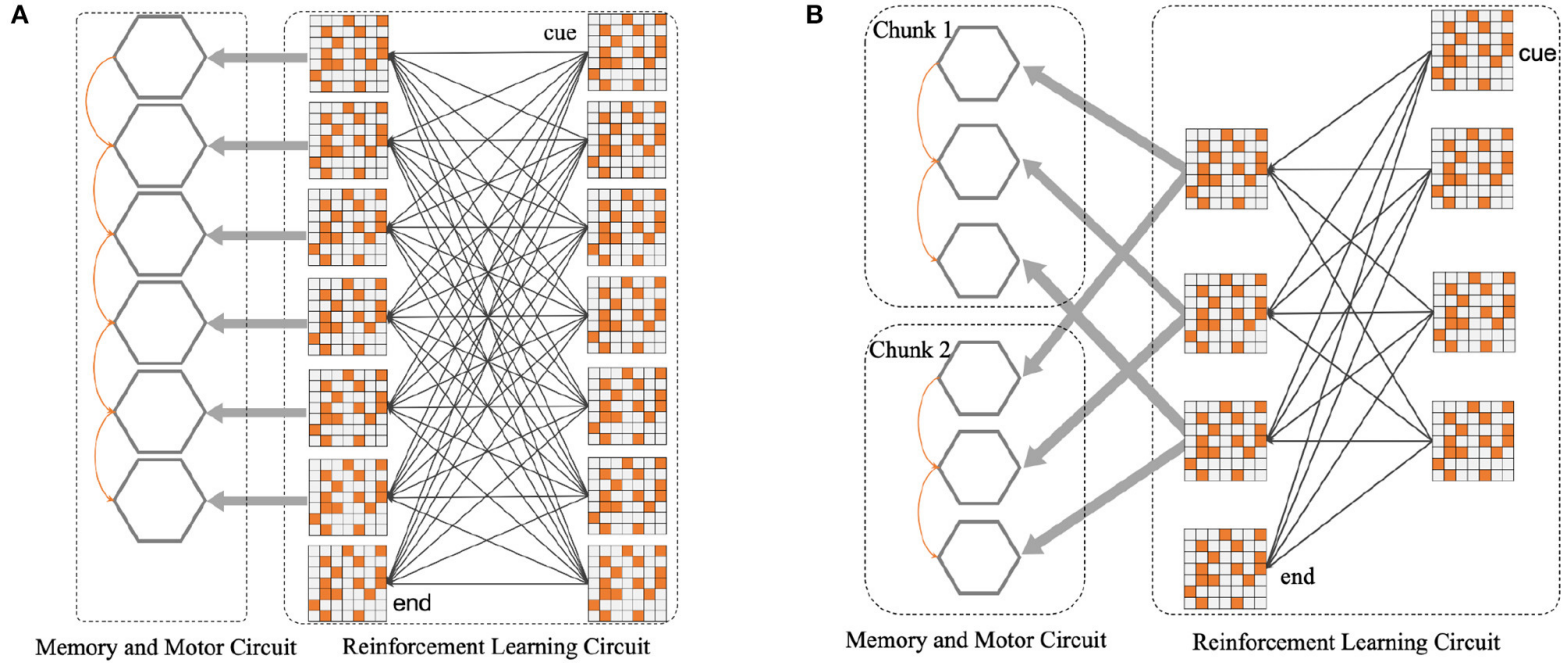
**(A)** Before the introduction of chunking strategy, the neural network architecture diagram. **(B)** After the introduction of chunking strategy, the neural network architecture diagram. For the convenience of composition, WMC and motor circuit are merged into gray hexagons, each refers to six different position symbols.

For instance, as shown in Figure 4A, several populations of neurons corresponding to six position symbols in the WMC fire sequentially according to the order in which symbols appear. The synaptic connections among six gray hexagons will be shaped by STDP rule, representing the memory for a 6-length sequence. Figure 4B shows how the network splitting a 6-length sequence into two 3-length chunks. Based on this instance, we completed the construction of spike neural network in the form of Figures 4A,B in the follow-up experiment, to explore the influence of chunking mechanism.

## 3. Experiment

### 3.1. Sequence Memory With Population Coding and STDP

First of all, we completed the construction of WMC, whose structure is shown in the Figure 1.

**Figure 5:**
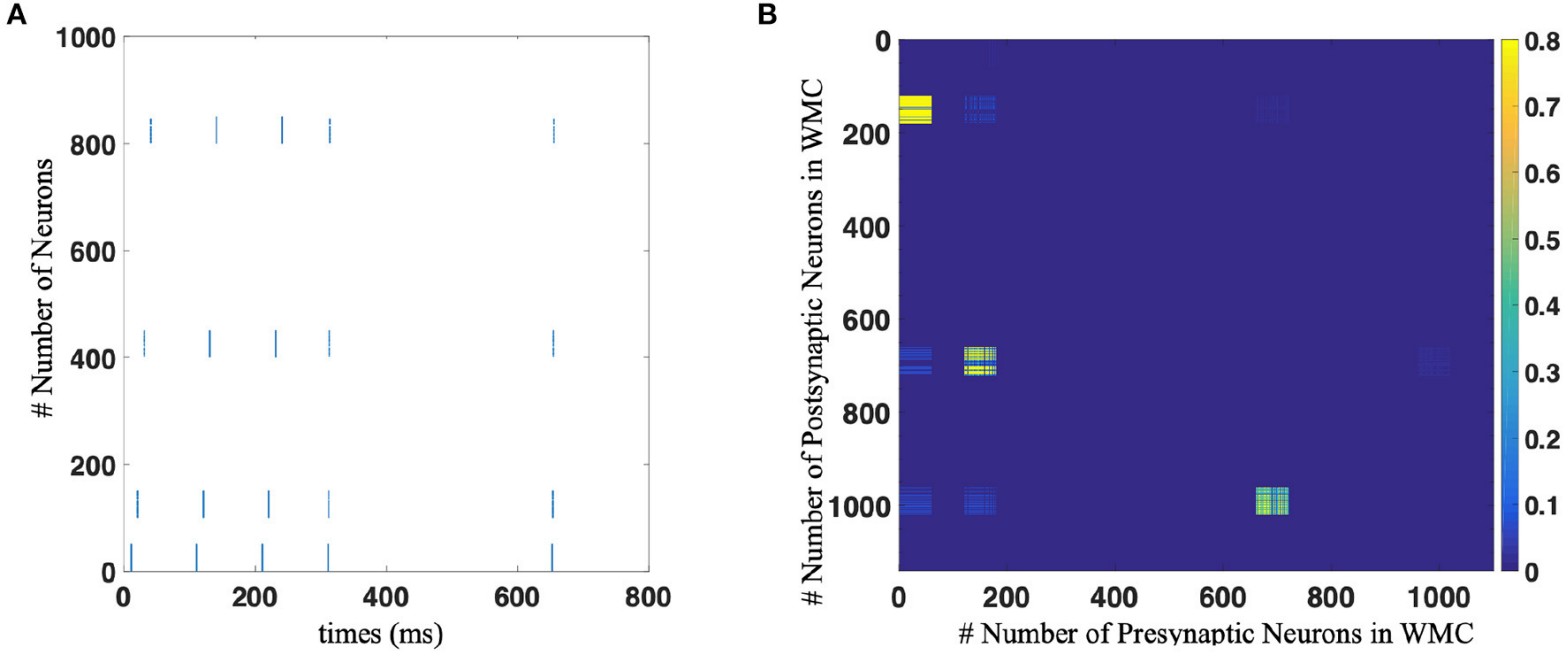
**(A)** The spike trains of neural network of working memory task with population coding strategy. Each blue dot indicates that the neurons corresponding to the vertical axis discharge at the time node corresponding to the horizontal axis. The number of neuron in population is 60 in this implementation. **(B)** The weights distribution of neural network after implement STDP learning.

The experimental process is divided into two stages: memory stage and test stage. In the memory stage, the original sequence was repeatedly displayed to SP-SNN several times. Each time a position symbol appears, the corresponding population of neurons is stimulated by external current stimuli and fire. Here we use the Poisson encode to activate the neuron population. The sequential firing of different populations of neurons combined with the STDP rule formed specific synaptic connections, thus forming the memory of specific sequence. This process is shown in Figure 5A for 0–400 ms.

In the test stage, as shown in Figure 5A for 600–800 ms, we only give the network a start signal, i.e., activate the “begin” neuron population, and then let the network independent work without any external stimulation, and observe the firing state of the network. When more than half of the neurons in a neuron population fired, it is considered that the network outputs corresponding symbol. Only when all the symbols in the sequence are output in the correct order can we think that the sequence is correctly memorized. Figure 5B shows the synaptic connection between neurons after one trial of sequence memory experiment.

In Working Memory Circuit (WMC), all connections are initialized to a small random number very close to 0. Neural plasticity occurs between different populations of neurons to realize sequence memory, and weak synaptic connections are maintained within the neuron population.

In order to better understand whether the population coding strategy contributes to the robustness of the model, we tested the recall accuracy of the WMC toward 3-length sequence with and without population coding strategy for different intensities of background noise, which is widely present in the human brain (Hidaka et al., 2000; Mišic et al., 2010). We introduce Gaussian white noise as the background noise of the network.Each neuron receives a stimulus current of Gaussian white noise, that is, a random variable with a mean value of 0 and a variance of σ^2^. According to the definition, the noise intensity of white Gaussian noise equals to σ^2^. We test the accuracy of the network under different noise intensity.

**Figure 6:**
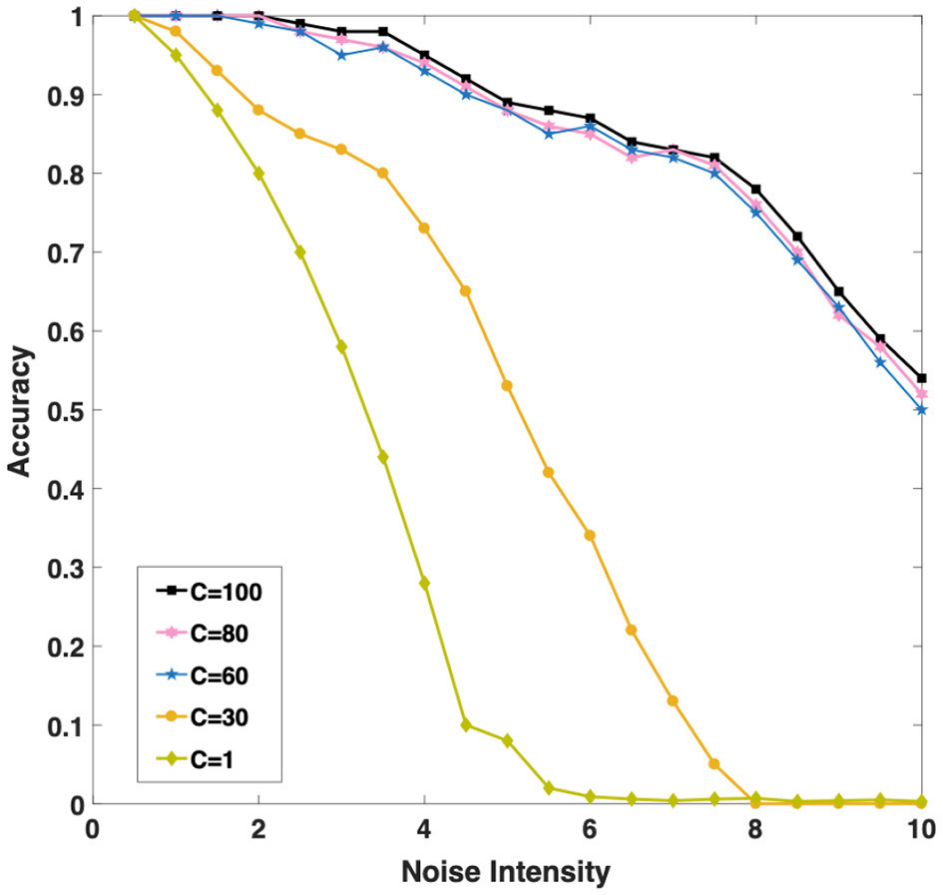
Three different color curves show that the memory accuracy of the network for the sequence decreases with the background noise enhancement when *C* (the number of single neurons population) takes different values.

Intuitively, as Figure 6 shown, we found with the increase of *C* (the number of single neurons population), the noise resistance of the model becomes more robust. When we increase *C* from 1 to 30 and then to 60, the accuracy of the model is greatly improved under the same noise conditions. Population coding strategy can indeed significantly improve the robustness of the model. However, when *C* continues to increase to 80 or even 100, the accuracy of the model increases slightly. It is interesting to explore the cause, we will discuss this phenomenon in the next chapter. Considering the computational complexity and experiment, we set *C* = 60 to carry out the follow-up experiments.

### 3.2. Sequence Production

**Figure 7:**
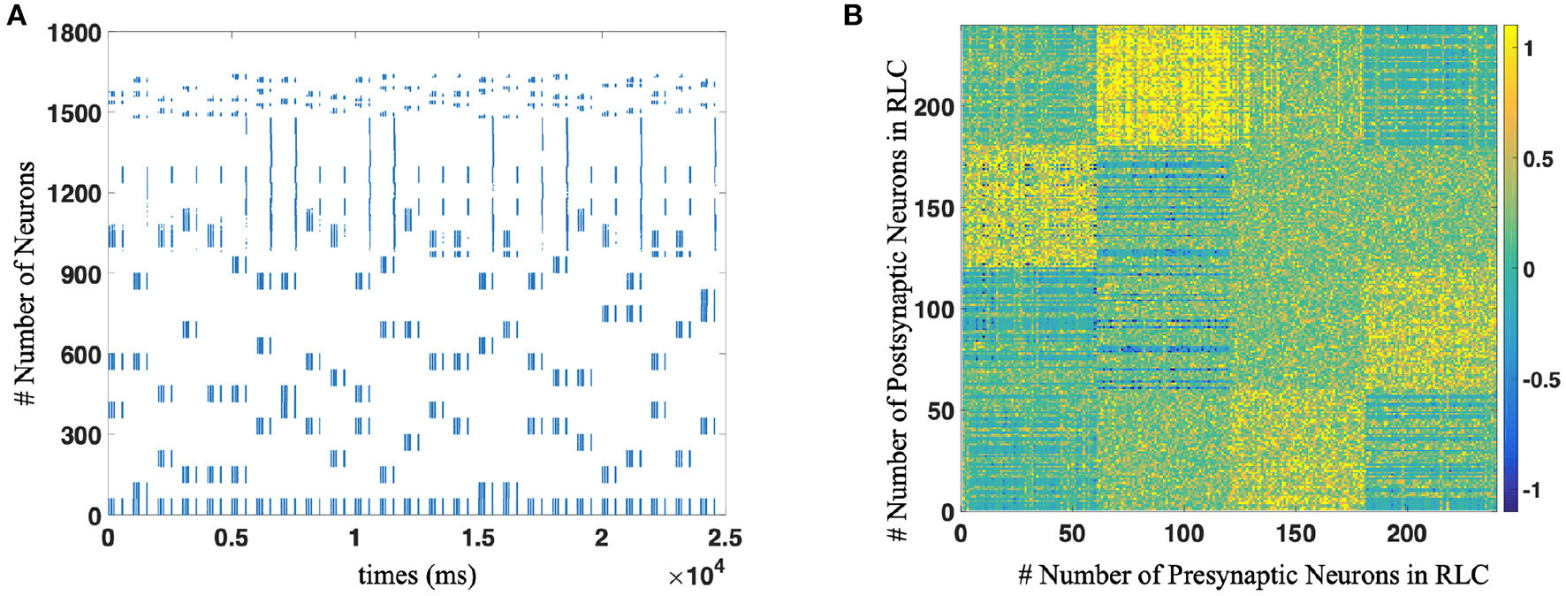
**(A)** The spike trains of the whole neural network during R-STDP training process. Each blue dot indicates that the neurons corresponding to the vertical axis discharge at the time node corresponding to the horizontal axis. As for the number of neurons, WMC is composed of No. 1-979 neurons, No. 980–997 are motor neurons and RLC consists of No. 998 1639 neurons, respectively. **(B)** The weights distribution of Reinforcement Learning Circuit after implement R-STDP learning, which contains the supra-regular grammars (Mirror rule in this figure).

Then, we completed the whole network test, including WMC, MC, and RLC, and demonstrated that SNN could reproduce the sequence according to different rules. For example, for the reverse production of a sequence of length 3, the spike trains and the strength of synaptic connections between different populations of neurons are shown in the Figure 7.

Compared with the result in Jiang et al. (2018), they found that the accuracy of macaque monkeys in the process of production sequences according to Mirror rules after the acquisition of rules is U-shaped as Figure 5A in Jiang et al. (2018) shown. Neuroscientists claim that the main reason causes this phenomenon are: the superposition of primacy and recency effect, which are considered essential in the process of evolution (Luchins, 1957; Jiang et al., 2018).

It is a well-established finding that the items at the beginning or at the end of the list are more likely to be recalled than the items in the middle of that list, which are termed the primacy and recency effects (Stewart et al., 2004). And both primacy and recency effects can be obtained in nonhuman primates (Castro and Larsen, 1992).

**Figure 8:**
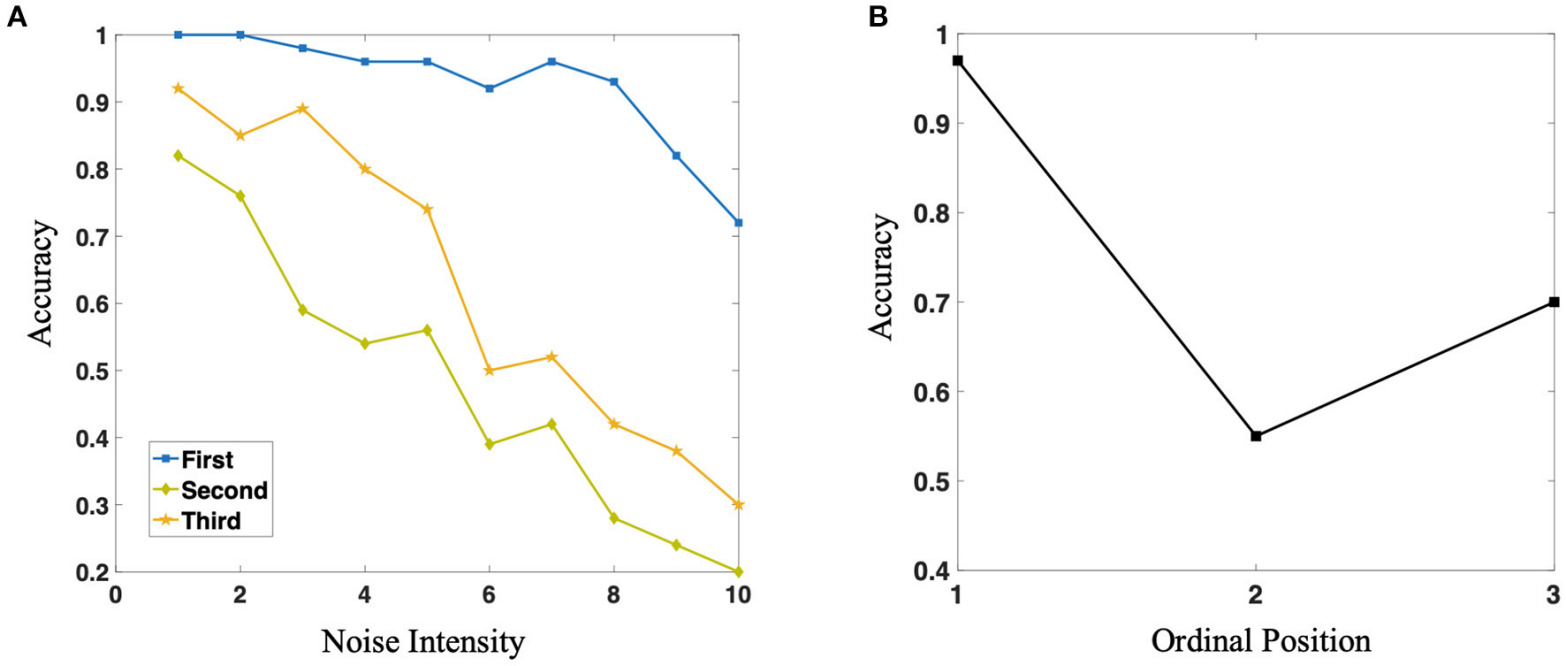
**(A)** Three different color curves represent the reconstruction accuracy of the network for three different positions in the sequence, respectively. With the increase of noise intensity, the accuracy of the three positions show a downward trend, but they always maintain the relationship of U-shape. **(B)** The average value of production accuracy of neural network for three different positions under different noise intensity.

In our practical simulation experiment, we found that the accuracy of the production of different position symbols is close to 100% after the network acquires specific rules, which is difficult to reflect the difference of different positions in the sequence. Therefore, in order to compare with biological experiments, we pull-in the background noise based on the original network. What we are very excited about is: as shown in Figure 8A, with the increase of noise, the accuracy of different position symbols in the network production sequence is gradually decreasing, but the production accuracy of different position symbols in the sequence has always maintained this U-shaped structure, which shows that the network structure and connection structure we constructed is highly biologically interpretable. Furthermore, Figure 8B shows the average accuracy of three positions. It is of some enlightenment to further understand how macaque monkeys can complete the task of sequence production and break the grammatical barrier.

### 3.3. Sequence Production With Chunking Mechanism

For the network structure after the introduction of Chunking Mechanism, since the production rules are consistent within each chunk, different chunks can share a population of reinforcement learning postsynaptic neurons, as shown in Figure 4B.

**Figure 9:**
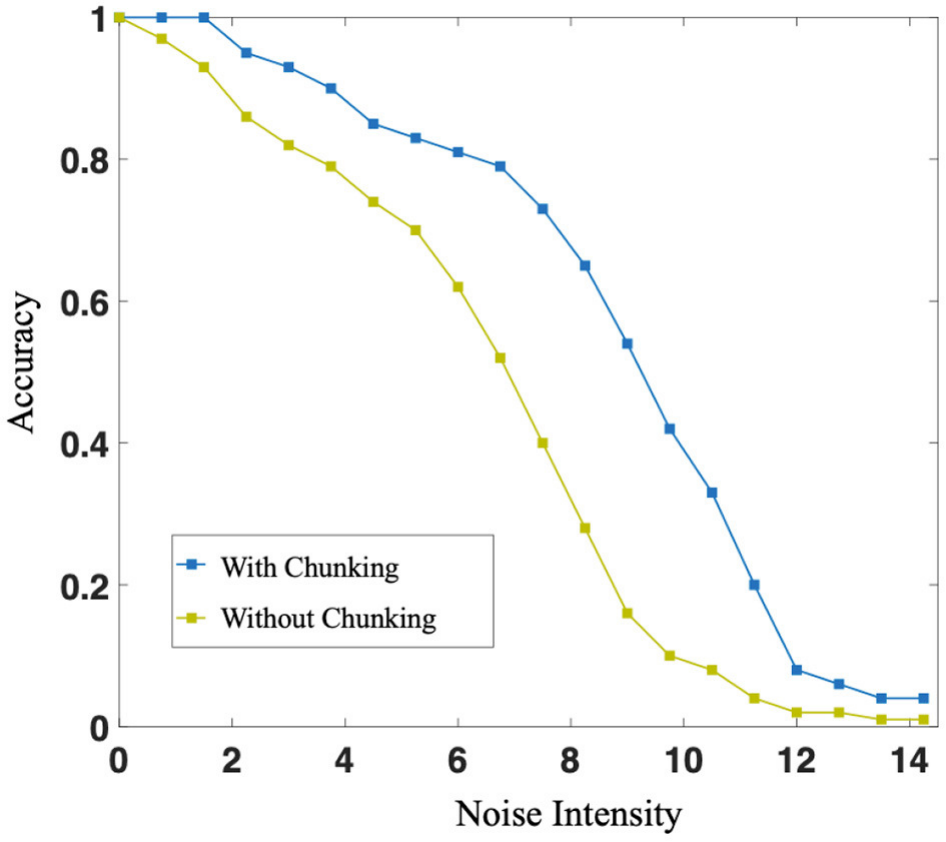
The blue curve and the green curve represent the production accuracy of neural network with or without chunking strategy, respectively. With the increase of noise intensity, both accuracy decreases. However, the accuracy of networks with chunking strategy is always higher than that without chunking strategy.

Individually, in this case, in the sequence memory stage, six symbols appear sequentially and are cut into two chunks of 3+3. Two chunks form specific connections to complete local memory. In the sequence production stage, the RLC begins to work, and in conjunction with the WMC, correct motor neurons fire, completing the so-called sequence production. During the experiment, we trained the original network, as shown in Figure 4A, and the network after introducing Chunking Mechanism, as shown in Figure 4B, respectively. Then, we compared the difference in production accuracy between the two networks under different noise conditions, the result is shown in Figure 9. As the noise intensity increases, the accuracy of both networks decreases. However, the accuracy of Chunking Mechanism network is always higher than that of the original network, which fully reflects the vital role Chunking Mechanism plays in this task. We will discuss the reasons for Chunking Mechanism's role in detail in section 4.

## 4. Discussion

We demonstrated that through the fusion of neuron population coding, STDP synaptic learning mechanism, and reinforcement learning mechanism (R-STDP), the SNN network could perform the same ability as macaque monkeys to construct the sequences according to super-regular rules, which was previously considered unique to humans. As far as we know, our work is the first to complete the research of sequence production based on SNN in accordance with supra-regular rules at the computational level.

Inspired by the research about “grandmother cells” in neuroscience (Bowers, 2009; Quiroga, 2012), we proposed to use the activation of a population of neurons to represent the emergence of a specific symbol in the brain, which may be caused by extra stimuli (correspond with the memory stage), or by the current from other populations of neurons within the network (correspond with the sequence production stage). In the process of experiment, we found that the population coding has stronger robustness and stability than the single neuron coding. However, when the number of neurons in the group reaches a certain degree, the robustness of the network tends to be stable and will not grow infinitely. How the brain sets the size of neuron populations to balance the robustness and consumption will be a very interesting topic in our future research.

Inspired by the training process toward macaque monkeys in the experimental paradigm of sequence production (Jiang et al., 2018), we introduced the reinforcement learning mechanism in super-regular rule learning, mainly using the R-STDP mechanism with eligibility traces method (Frémaux and Gerstner, 2016). Before defining the structure, we thought the difficulty of this work is that every time a network (or monkey) gets a reward or punishment, it is easy for the network (or monkey) to link the symbols with the reward and punishment signals, actually the production rules behind the symbols are related to the reward and punishment signals. In our experiments, we substantially helped the network to complete the transition from symbols to rules behind symbols, that was, reward and punishment signals are associated with rules, not just with symbols themselves. In the future work, how to let the network automatically complete this process, rather than directly tell the network in a priori way, is a very worthy of study.

In the experimental results of Jiang et al. (2018), the accuracy of symbol production for different positions in the sequence is different, and generally presents a U-shaped rule, i.e., the effect of sequence production in the middle of the sequence is weaker than that at the beginning and the end. This feature is considered to be an essential feature in the evolution process. Psychologists and cognitive scientists believe that this phenomenon is the result of the superposition of the primacy and recency effect (Luchins, 1957; Stewart et al., 2004).

During the experiment, our proposed network structure was consistent with macro-cognitive behavior of macaque monkeys, showing the accuracy of U-type production. The reason why the network can show U-type accuracy is that our proposed network structure combined the “primary effect” and “recency effect” simultaneously.

Respectively, as for the “primary effect,” in WMC, the neuron populations corresponding to the first symbol in the sequence is stimulated by the “begin” neuron population, and most of the neurons in the “begin” neuron population are firing in the specific time window belong to “begin” population, which leads to the firing of the first symbol neuron population will be more intense, directly leading to the greater increase of membrane potential of the corresponding motor neuron of the first symbol. Therefore, the first symbol has better noise resistance, causing the so-called “primary effect.”

About the “recency effect,” because the building block of spike neural network is a LIF neuron model, and the LIF neuron model has a leakage mechanism (Miller, 2018), the membrane potential of the pre-activated motor neuron gradually decreases with the passage of time, and the last sign appears because of its shortest production time, and its membrane potential decreases the least, resulting in the “proximal effect.”

Inspired by Chunking Mechanism in the biological brain, we implemented the Chunking Mechanism based on SNN in the experiment and verified that it plays a vital role in improving the accuracy of production. After analysis, we found that Chunking Mechanism can shorten the sequence length of the production process. In the example of Figure 4, the original network without Chunking Mechanism needs to recall the whole sequence of length six first, and then produce it with RLC. Because of the leakage characteristics of motor neurons, the membrane potential of motor neurons at corresponding locations decreases dramatically, which makes it easier to make mistakes. While Chunking Mechanism is introduced, hardly when every chunk is recalled, it will be produced immediately. Compared to the original network, the duration of the decline of the membrane potential of the new network motor neurons will be shorter, and the corresponding membrane potential will be higher, causing the higher accuracy.

However, we must admit that the chunking mechanism used in this work still has some limitations. The main limitation is that we implicitly help SP-SNN to divide a long sequence into several chunks, that is, to decompose a sequence of length six into two subsequences of length 3. However, in the actual human cognitive process, cutting long sequences into chunks has substantial autonomy and flexibility. How can SNN complete this process spontaneously? How do different segmentation methods, such as equal-length segmentation and unequal-length segmentation, affect the cognitive process's results? These are the problems worthy of exploration in the future. However, our work still completes the preliminary exploration of the chunking mechanism and demonstrates that the chunking mechanism is of great help to improve the model's robustness.

The following will discuss the difference between our work and the current popular artificial neural network or deep learning. The difference mainly consists of three parts.

First, almost all the current artificial neural networks set the weight between neurons to be fixed at the inference stage (only changes when the network is training), which is different from the real nervous system. In the real nervous system, the connection between neurons will be affected by the strength of input signal, time process and other factors, temporary change with neuron activity, which is called short-term plasticity of the synapse (STP), also known as dynamic connection (Markram and Tsodyks, 1996; Fung et al., 2015).

From the computational point of view, STP provides the biological neural network one more time dimension in information processing than the artificial neural network with a fixed weight, so it has more computational potential and can perform complex cognitive tasks. From this point of view, it is obvious that in our network WMC adopts short-term plasticity (STP). While the synapse in RLC changes in the long term, which can be looked like a particular kind of long-term plasticity (LTP). Compared with artificial neural network, our network integrates STP and LTP mechanism, makes full use of time dimension, and to a certain extent, expands the boundary of SNN's information processing capacity.

Second, although the neural network trained by the current deep learning technology can solve some specific problems, its plausibility is very poor (Castelvecchi, 2016), which leads to serious security problems (such as Adversarial Examples Problem) and becomes a black cloud over the head of deep learning (Goodfellow et al., 2014; Liu et al., 2016).

However, our model is totally different. In the experiment, we can check the firing state and weight distribution of network at any time, which can be used to judge the working memory of the current network, the learning process of rules, and so on. That is to say, the network we build is completely interpretable. Although the complexity of our model is not comparable to the current deep learning technology, our work may bring some inspiration for the construction of more interpretable artificial intelligence system in the future.

Finally, for researchers in the field of neuroscience and cognitive science, our work provides a new perspective to some extent, that is, how to use the neural network of connectionism to represent the symbol reasoning of symbolism. In this work, we demonstrate the feasibility of using spike neural network to complete the task of production sequence according to supra-regular rules, breaking the “syntax barrier” of animals. For further explore the representation of symbols in the animal brain, as well as how non-human primates such as macaque monkeys complete the task of sequence representation, our work lay the foundation of computing.

## 5. Conclusion

This paper proposed a Brain-inspired Sequence Production Spiking Neural Network (SP-SNN) to model the Sequence Production process, inspired by a biological experiment paradigm which showed that macaques could be trained to produce embedded sequences involved supra-regular grammars.

After experimental verification, we demonstrated that SP-SNN could also handle embedded sequence production tasks, striding over the “syntax barrier.” SP-SNN coordinates STP and LTP mechanisms simultaneously. As for STP, Population-Coding and STDP mechanisms realize working memory. As for LTP, the R-STDP mechanism shapes Reinforcement Learning Circuit for different supra-regular grammars, whose synaptic weights do not change until a reward/punishment occurs. The U-shape accuracy of the results of SP-SNN and macaque, which is caused by the superposition of primacy and recency effect, further strengthened the biological plausibility of SP-SNN. Besides, we found the chunking mechanism, i.e., divides a long sequence into several subsequences, indeed makes a difference to improve our model's robustness.

As far as we know, our work is the first one toward the “syntax barrier” in the SNN field. In future research, we hope to compare the electrical activity of SP-SNN with the electrophysiological data of macaque in the sequence production task to expose more underlying animals' neural mechanisms in this cognitive process.

## Data Availability Statement

The original contributions presented in the study are included in the article/supplementary material, further inquiries can be directed to the corresponding author/s.

## Author Contributions

HF and YZ designed the study and performed the experiments. HF, YZ, and FZ developed the algorithm, performed the analysis of the results, and wrote the manuscript. All authors contributed to the article and approved the submitted version.

## Conflict of Interest

The authors declare that the research was conducted in the absence of any commercial or financial relationships that could be construed as a potential conflict of interest.
